# Molecular prevalence and associated infection risk factors of tick-borne protozoan and rickettsial blood pathogens in small ruminants

**DOI:** 10.1186/s12917-023-03702-4

**Published:** 2023-08-31

**Authors:** Mennat-Allah Abdelsalam, Wael Felefel, Sabreen Fadl, Mohamed Bessat

**Affiliations:** 1 Department of Parasitology, Faculty of Veterinary Medicine, Matrouh University, Matrouh, Egypt; 2 Department of Biochemistry, Faculty of Veterinary Medicine, Matrouh University, Matrouh, Egypt; 3 Department of Parasitology, Faculty of Veterinary Medicine, Alexandria University, Alexandria, Egypt; 4 Faculty of Veterinary Medicine, King Salman International University KSIU, Ras Sudr, South Sinai, Egypt

**Keywords:** Molecular epidemiology, Phylogenetic analysis, Tick-borne blood pathogen, Small ruminants

## Abstract

**Background:**

Tick-borne blood pathogens cause highly pathogenic diseases, which are associated with substantial economic losses in ruminants. Despite this, epidemiological research on these pathogens remains neglected in many countries. This study initiated a regional epidemiological survey that included the detection of molecular prevalence, associated risk factors, and gene sequencing, combined with phylogenetic analysis, targeting the two main tick-borne blood protozoan and rickettsial pathogens of *Babesia, Theileria, and Anaplasma* that infect small ruminants. One hundred blood samples were collected from 76 sheep and 24 goats.

**Results:**

Microscopic examination of Giemsa-stained blood films revealed that 73% of the samples were infected with at least one species of the three blood pathogenic organisms. Molecular diagnosis based on the 18 S rRNA for *Babesia* and *Theileria* species and the major surface protein 4 (msp4) for *Anaplasma* species, revealed that 43% of the small ruminants were infected with at least one of these pathogens. The animal’s sex was the most significant associated risk factor, with 49.4% of female animals infected compared with only 4% of male animals (*P* < 0.05). The open breeding system recorded the highest infection rate for tick-borne blood pathogens. Homology-based and phylogenetic analyses indicated that the specific isolate species were *Babesia ovis* (*B. ovis*), *Theileria ovis* (*T. ovis*), and *Anaplasma ovis* (*A. ovis*), with sequences showing significant identities with isolates from sheep, goats, and other animal species, and geographically diverse countries in Africa, Asia, and Europe, in addition to Egypt.

**Conclusion:**

This was the first molecular evidence of *B. ovis, T. ovis*, and *A. ovis* infections in sheep and goat populations in the North Coast region of Egypt. More extensive studies are required to develop an epidemiological map of blood pathogenic organisms, while more effective control strategies are required to reduce the burden of tick-borne pathogens on small ruminants.

## Background

Small ruminants play an important role in the survival, economy, and social livelihoods of people throughout the world. More significantly, in developing countries, keeping small ruminants is crucial to meeting people’s needs for food, specifically meat and milk. Their wool is also used in many industries that contribute to national economies [1].

Infections of small ruminants by the piroplasmid and rickettsial blood pathogens, including *Anaplasma (A.) ovis, A. bovis, (A) phagocytophilum, Babesia (B.). motasi-like, (B) motasi-like Xinjiang, Theileria (T.). lestoquardi, T. uilenbergi, and T. luwenshuni* have direct effects, including debilitating health conditions that are primarily attributable to hemolytic anemia and decreased feed conversion ratio. If not treated on time, progressive parasitic infections will ultimately result in morbidities and high mortality rates among infected animals [2, 3]. Parasitic and rickettsial blood infections are especially challenging due to their unknown prevalence rates, various modes of transmission, and the increasing resistance to commonly applied anti-parasitic chemotherapies [4].

Babesiosis in small ruminants is caused by various *Babesia* species, but the two most responsible species are *Babesia motasi* and *Babesia ovis* [5]. In contrast, ovine theileriosis is caused by several *Theileria* species, which vary in their infectivity and pathogenicity. *Theileria lestoquardi* is the most pathogenic, while *Theileria ovis* is the least pathogenic [6]. Anaplasmosis in small ruminants, on the other hand, is mostly caused by one of three species, *Anaplasma ovis*, *Anaplasma phagocytophilum*, and *Anaplasma bovis* [7, 8]. Worldwide, *A. ovis* is the most prevalent *Anaplasma* species in small ruminants [8] and is also the main causative agent of ovine anaplasmosis in tropical and subtropical regions [9].

Under field conditions, the diagnosis of tick-borne blood pathogens depends primarily on collecting case history data, identifying clinical symptoms in animals, and performing laboratory diagnoses [10, 11]. Laboratory diagnosis primarily includes microscopic examination of Giemsa-stained blood smears to detect erythrocytic stages of protozoan blood parasites and molecular detection of parasitic agents [10, 12]. Molecular techniques such as polymerase chain reaction (PCR), restriction fragment length polymorphism, and phylogenetic analysis are also applied to detect previously unidentified species of disease-causative protozoan parasites and are also used to differentiate between different species of the same genus in different hosts and different districts [10].

The current study was initiated to identify tick-borne blood pathogens infecting sheep and goats located in the northern coastal region of Egypt utilizing microscopic, molecular, and phylogenetic analysis techniques. During the identification process, risk factors and their potential associations with tick-borne blood pathogens infections were also analyzed.

## Results

### Demographic data of the study animals

The majority of the sampled animals were sheep (76%), compared with goats (24%), while female animals predominated in the sampling group over males (79% versus 21%) (Table 1). Additionally, most of the sampled animals were older (≥ 3 years), while 26% were medium-aged animals (1–3 years), and the remaining animals were younger than 1 year old (16%). When grouped according to grazing practices, 62% were free grazing compared with 38% that were maintained in semi-closed grazing systems. Most of the sampled animals generally had good body scores (70%). Among the sample animals, the mucous membrane was normal in 32%, pale in 22%, and congested in 46%. Only 10% of the animals were feverish at the time of sampling, while 33% and 67% were recorded with or without the presence of ticks on animals, respectively (Table 1).

**Table 1:** Demographic data of small ruminants enrolled in the current study

Parameters		%
Animal Species	Sheep	76
	Goat	24
Sex	Male	21
	Female	79
Grazing System	Open	62
	Semi-closed	38
General body condition	High score	70
	Low score	30
Mucous Membrane	Normal	32
	Pale	22
	Congested	46
Temperature	Normal	90
	Feverish	10
Presence of ticks	Yes	33
	No	67
Age	≤ 1 year	16
	1–3 years	26
	≥ 3 years	58

.

### Microscopic detection

Microscopic examination of the 100 Giemsa-stained blood smear samples (76 samples from sheep and 24 samples from goats) revealed that 73% of the samples were infected with at least one species of tick-borne pathogens including the piroplasmid protozoan parasites of genera *Babesia* and *Theileria*, and that of rickettsial tick-borne *Anaplasma*. When analyzed, the rates of infection with *Babesia*, *Theileria*, and *Anaplasma* were 17%, 30%, and 65%, respectively.

### Molecular identification

PCR was successfully applied to amplify specific gene fragments from the three blood pathogens, with the expected molecular masses for the PCR products of *B. ovis*, *T. ovis*, and *A. ovis* being 549, 509, and 870 bp, respectively. Data obtained revealed that 43 out of 100 samples were positive for at least one of the three tick-borne blood pathogens in the four selected districts of the Matrouh Governorate (Table 2). *Babesia ovis*, *T. ovis*, and *A. ovis* were recorded in 16%, 17%, and 24% of the total examined samples, respectively. The molecular prevalence data were used in all analyses from now and henceforth.

**Table 2:** Molecular prevalence of *Babesia, Theileria, and Anaplasma* among the four selected districts (Elhammam, Marsa-Matrouh, Eldabaa, and Elalamein) in Matrouh Governorate

		PCR diagnosis as a screen test		Total Number of Samples	Monte Carlo Sig. (2-sided)		Logistic Regression 95.0% C.I.	Sig
		Yes	no		X^2^	P		
Area locality	Elhammam	11	21	32			1.050(0.373–2.959)	0.926
	Eldabaa	17	14	31	4.738	0.198	0.453(0.163–1.257)	0.128
	Elalamein	4	2	6			0.275(0.043–1.749)	0.171
	Matrouh	11	20	31			Reference	
**Total**		**43**	**57**	**100**				

.

### Distribution of infection of *B. ovis*, *T. ovis*, and *A. ovis* among the four selected districts of matrouh governorate

Data showed that no significant differences (X2 = 4.738, P = 0.198) were detected between the four studied areas, concerning the distribution of infection by the three blood pathogens (Table 2). When analyzed as risk factors, Elhammam district was considered a higher risk factor when compared with the reference area (Matrouh district), with odds Logistic Regression 95% CI 1.050 (0.373–2.959). Moreover, Table (3) showed that the association between infection of *B. ovis*, *T. ovis*, and *A. ovis* species and animal species (sheep and goats) was insignificant, with only the more risk of getting an infection with *Babesia* and *Anaplasma* among sheep than goats, with the Odds Ratio 95% Confidence Interval (1.444 (0.375–5.566) and 1.267(0.416–3.857, respectively).

**Table 3:** The molecular prevalence of babesia, theileria, and anaplasma obtained among the different animal groups in matrouh governorate

Blood Parasite		Animal species		^ Fisher’s Exact Test ^a^ Pearson Chi-Square		Odds Ratio 95% Confidence Interval
		Sheep	Goat	χ2	P value	
Detection of *Babesia* by PCR	Yes	13	3	0.174^a^	0.755^	1.444 (0.375–5.566)
	No	63	21			
Detection of *Theileria* by PCR	Yes	10	7		0.115^	0.368 (0.122–1.109)
	No	66	17			
Detection of *Anaplasma* by PCR	Yes	19	5		0.677	1.267 (0.416–3.857)
	No	57	19			

### Analysis of infection prevalence and associated epidemiological risk factors

Infections with tick-borne pathogenic organisms were recorded in 42.1% of sheep and 45.8% of goats, with no significant species-dependent differential, as shown in (Table 4). The second and only significant factor observed was animal sex, with 49.4% of females infected, compared with only 4% of male animals (*P* < 0.05). A higher infection rate of 48% was observed in older animals (≥ 3 years) compared with medium-aged and young animals, in which 42.3% and 25% infection rates were observed, respectively (Table 4).

**Table 4:** Analysis of the epidemiological risk factors and its association with blood parasites

Parameters	No Examined	Infected (%)	OR (95% *CI*)		*p*-value
Species					
Sheep	76	42.10%	0.860 (0.341–2.163)		0.748
Goat	24	45.80%			
Sex					
Male	21	19.00	0.241(0.075–0.782)		0.013
Female	79	49.40			
Age group					
≤ 1 Y	16	25.0	2.904 (0.794–10.618)		0 .249
1–3 Y	26	42.3	1.158 (0 .411–3.261)		
≥ 3Y	58	48.3	References		
Grazing system					
Open	62	45.20	1.263 (0.556–2.869)		0.577
Semi-closed	38	39.50			
General body condition					
High score	70	38.60	0.549 (0.232–1.303)		0.172
Low score	30	53.30			
Temperature					
Normal	90	42.2	0 .731 (0.198–2.704)		0.741
Feverish	10	50.0			
Presence of ectoparasites					
Yes	33	54.5	2.016 (0.866–4.695)		0.102
No	67	37.3			
Mucous membrane					0 .457
Normal	32	56.20		0.992 (0.349–2.819)	
Pale	22	68.20		1.734 (0.523–5.747)	
Congested	46	52.20		Reference	

The open grazing system was found to be more favorable to infection than the semi-closed one, with 45.2% and 39.5% infection rates, respectively. The remaining factors were found to have differential effects on infection rates, although with non-significant association patterns. Animals with high-score body conditions recorded lower infection rates (38.6%) than weak and emaciated animals (53.3%). Congested eye mucous membranes were associated with a 47.8% infection rate, while normal and pale membranes were associated with lower infection rates of 43.8% and 31.8%, respectively. When the animal body temperature was examined, 50% of animals were found to be infected, compared with 42.2% of animals with normal body temperatures. Finally, 54.5% of animals in which ticks were detected during sampling were found to be infected with blood pathogens, compared with a 37.3% infection rate in tick-free animals (Table 4).

### DNA sequencing and analysis

Homology analysis performed using GenBank sequence data revealed highly significant scores of identities, similarities, query coverage, and E. values, with top hits for highly homologous sequences associated with each of the two identified blood protozoon and rickettsial pathogens. Homology-based data confirmed the identities of the current isolates as *B. ovis*, *(A) ovis*, and *T. ovis*, respectively. The nucleotide sequences of *(B) ovis*, *T. ovis*, and *A. ovis* isolates in the current study were recorded in GenBank under accession numbers MZ801782.1, MZ801783.1, and MZ889099, respectively.

The current *B. ovis* isolate was well-aligned and highly homologous with *B. ovis* isolates from sheep, goats, and horses, and countries as diverse as Uganda, Turkey, and Spain, with identities of > 94% (Table 5). Albeit with slightly lower identities, other *Babesia* species, such as *Babesia orientalis*, *Babesia bigemina*, and *Babesia divergens* from China, Iraq, and Ireland, respectively, were also highly homologous. Top homology hits of *T. ovis* included isolates of *T. ovis* from sheep and donkeys located in Iran, Pakistan, and Egypt, with > 92% identities (Table 5). Similarly, *Theileria parva*, *Theileria lestoquardi*, and *Theileria orientalis*, presented > 90% homology over their 18 S rRNA sequences. In contrast to *Babesia* and *Theileria*, all BLAST homologous outputs of *Anaplasma* 16 S rRNA were those of *A. ovis* isolates from Iran, Sudan, and Russia, with > 90% identities (Table 5).

**Table 5:** BLAST-Homology analysis of sequence output of *Babesia ovis*, *Theileria ovis*, and *Anaplasma ovis*

Detected pathogens	Accession no	Host	Country	Identity (%)	E. Value
*Babesia ovis*	MZ801782.1	Sheep	Egypt	100.00	0.0
*Babesia ovis*	MT114713.1	Goat	Uganda	94.61	0.0
*Babesia ovis*	MG569902.1	Horse	Turkey	94.61	0.0
*Babesia ovis*	AY150058.1	Goat	Spain	94.61	0.0
*Babesia orientalis*	MH208616.1	Tick	China	92.58	0.0
*Babesia bigemina*	MH356483.1	Buffalo	Iraq	92.36	0.0
*Babesia divergens*	LC477142	Cattle	Ireland	90.93	0.0
*Theileria ovis*	MZ801783.1	Sheep	Egypt	100	0.0
*Theileria ovis*	KC599236.1	Sheep	Iran	94.50	0.0
*Theileria ovis*	MT498784.1	Sheep	Pakistan	92.87	1e-174
*Theileria ovis*	MN625903.1	Donkey	Egypt	92.87	1e-174
*Theileria parva*	HQ684067.1	Buffalo	South Africa	94.07	8e-161
*Theileria lestoquardi*	GU726902.1	Sheep	Iran	90.70	3e-29
*Theileria orientalis*	LC576821.1	Cattle	Myanmar	93.75	2e-157
*Anaplasma ovis*	MZ889100.1	Sheep	Egypt	100	0.0
*Anaplasma ovis*	MH790274.1	Sheep	Iran	99.87	0.0
*Anaplasma ovis*	MF740823.1	Sheep	Sudan	99.8	0.0
*Anaplasma ovis*	MW535731.1	Cattle	Russia	99.74	0.0

### Phylogenetic analysis

Phylogenetic trees of *B. ovis* and *T. ovi*s were constructed based on 18 S rRNA gene sequences, and a phylogenetic tree of *A. ovis* was constructed based on msp4 gene sequences, which were generated in this study or downloaded from GenBank.

**Fig. 1:**
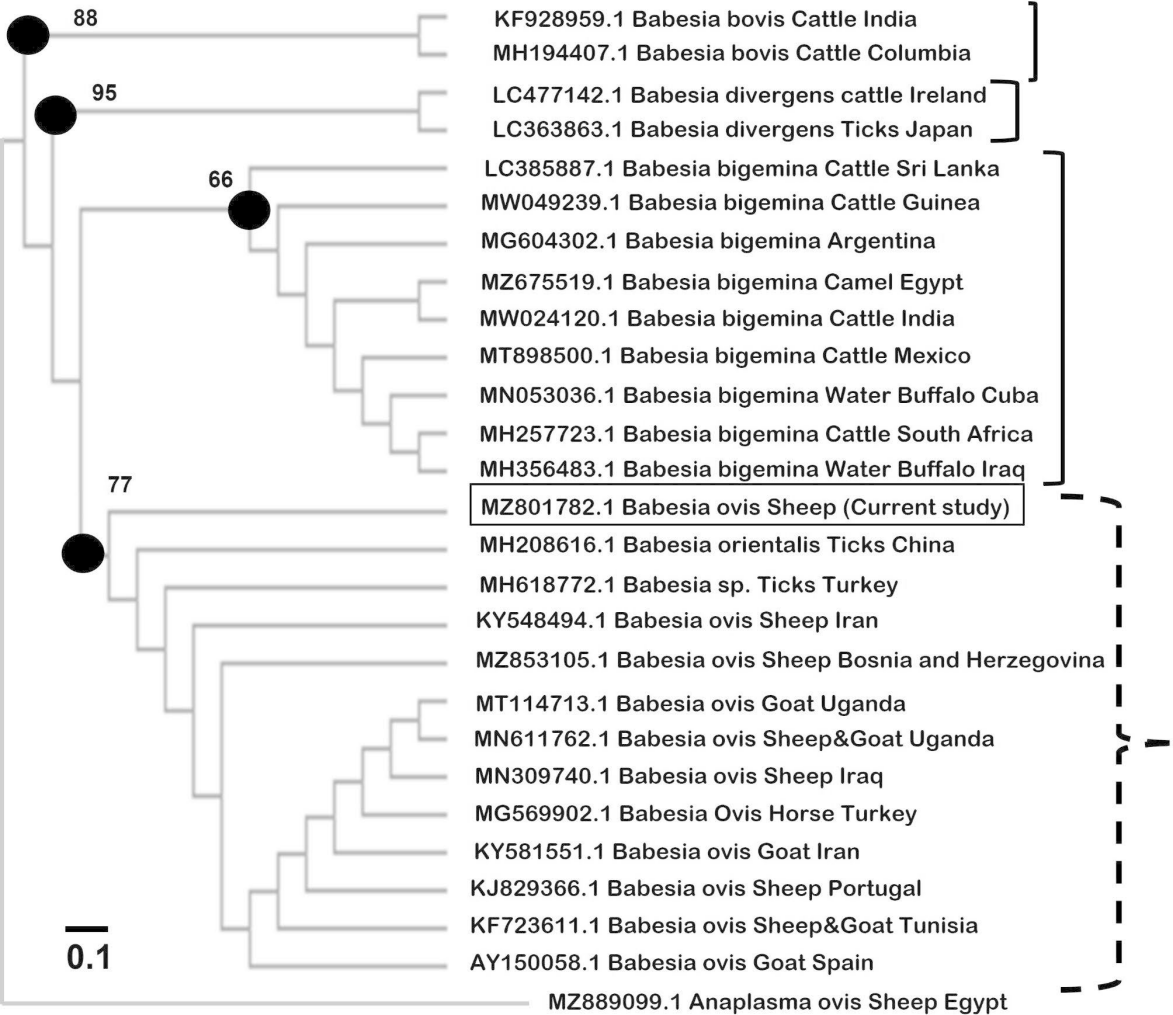
The dendrogram was constructed using the neighbor joining (NJ) method based on 18 S rRNA partial sequences of *Babesia ovis* isolate. The distance of similarities is based on the molecular basis evolution of minisatellite sequence regions in *Babesia ovis* genomic DNA. The new sequence reported in the current study is indicated (squared)

The neighbor-joining-based phylogenetic analysis of the 18 S rRNA nucleotide sequences of *Babesia* spp. revealed a highly conserved evolutionary relationship of current *B. ovis* as it has been grouped in the same clade with other related species from sheep, goats, horses, and ticks from various geographical locations (Fig. 1). The only exception was *B. orientalis* from ticks in China. Other *Babesia* species were systemically grouped into three specific clades, which were then grouped exclusively as *B. bigemina*, *Babesia bovis*, and *Babesia centralis*. These included isolates from different hosts and geographically diverse countries (Fig. 1).

**Fig. 2:**
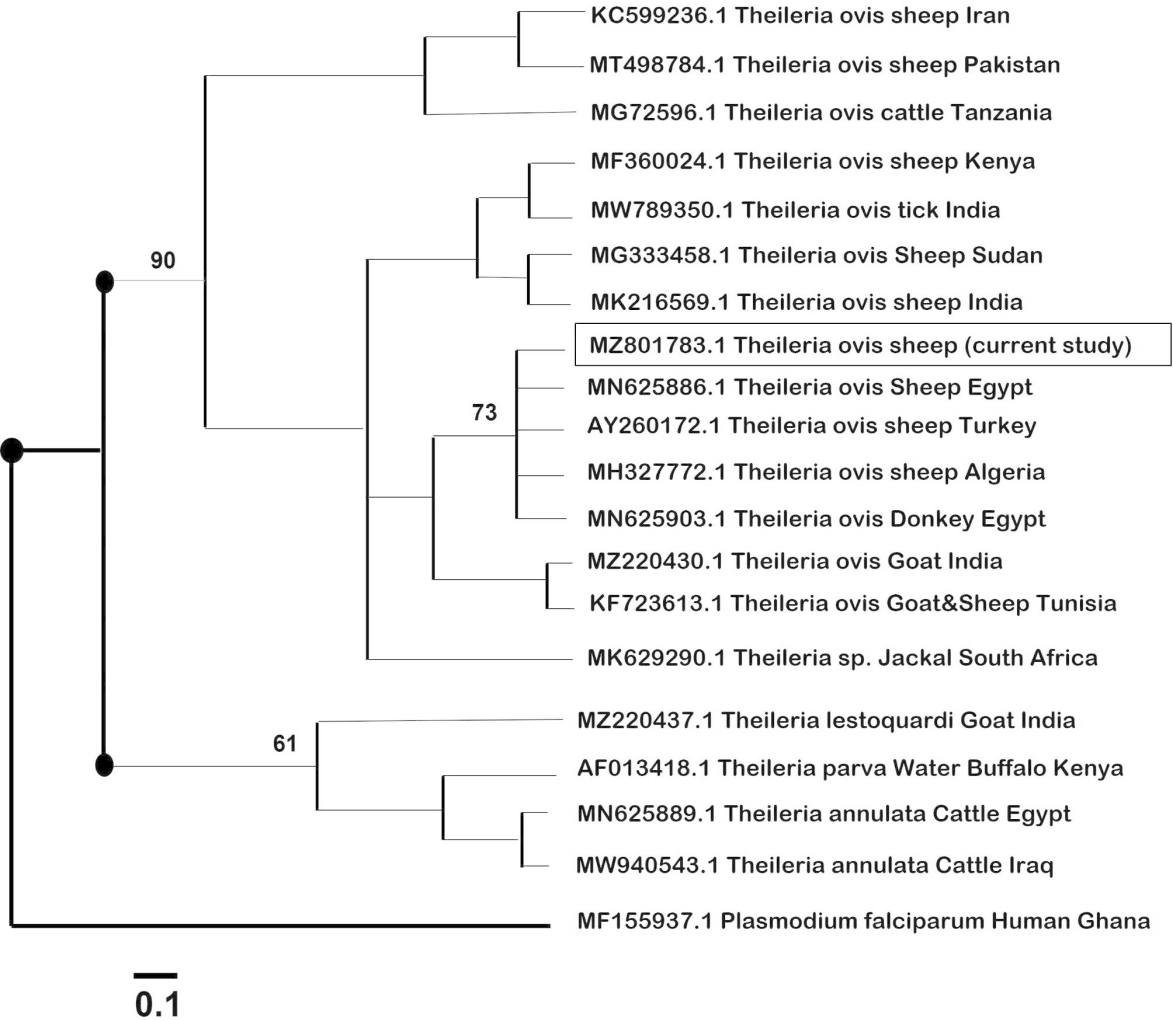
NJ-based phylogenetic analysis of 18 S rRNA partial sequences of the *Theileria ovis* isolate. The distance of similarities is based on the molecular basis evolution of minisatellite sequence regions in *T. ovis* genomic DNA. The new sequence has been similarly indicated (squared) on the tree branches

**Fig. 3:**
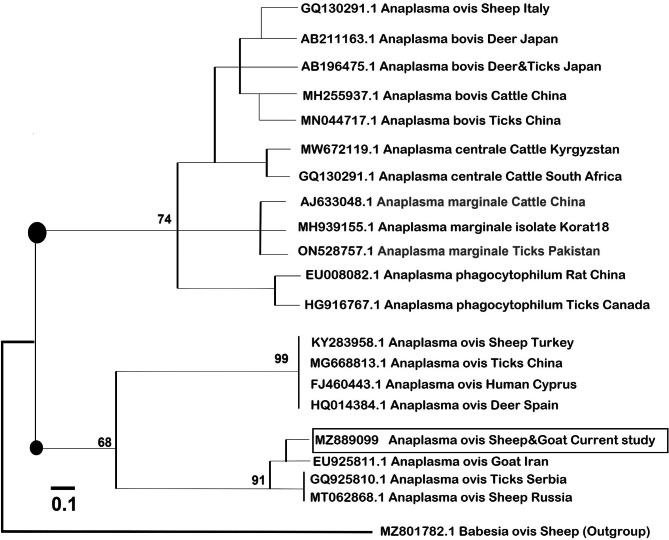
NJ-based phylogenetic analysis of the msp4 partial sequences of the *Anaplasma ovis* isolate. *A. ovis* sequence of the study has also been indicated. The distance of similarities is based on the molecular basis evolution of minisatellite sequence regions in *A. ovis* genomic DNA.

The phylogenetic analysis of partial 18 S rRNA nucleotide sequences of *T. ovis* resulted in two main clades, with *T. ovis* isolates being grouped into a large clade, and other species, such as *T. lestoquardi*, *T. parva*, and *Theileria annulata*, being grouped into another large clade (Fig. 2). The current isolates in the same clade as *T. ovis* are isolated from countries as diverse as Iran, Sudan, Serbia, China, Mongolia, Pakistan, and Russia (Fig. 2). The current isolate was primarily associated with *T. ovis* in sheep, goats, and donkeys in Egypt, Turkey, Algeria, India, and Tunisia. The evolutionary distant relationships among *Anaplasma* isolates were more homogenous as inferred from their NJ tree. *A. ovis* isolates were clustered into a single parental clade that was further subdivided into two branches, with one branch linking the current isolate with *A. ovis* from sheep, goats, and ticks from Russia, Iran, and Serbia, while the other branch grouped *A. ovis* from sheep, deer, ticks, and humans from Spain, Cyprus, China, and Turkey (Fig. 3). Species of *Anaplasma*, other than *A. ovis*, were remarkably grouped into unique clades with isolates of the same species, whether *A. bovis*, *A. centrale*, *Anaplasma marginale*, and *A. phagocytophilum*, and were clustered together regardless of their hosts or geographic origins (Fig. 3).

BLAST analysis of the 18 S rRNA identified the current isolate as *T. ovis*, and it was deposited in GenBank with accession number MZ801783.1. The *T. ovis* sequence of the current study revealed high homology-based identities of 94.5% and 92.87% with *T. ovis* genotypes from sheep from Iran and Pakistan (KC599236.1, MT498784.1), respectively), while a *T. ovis* isolate from donkeys (MN625903.1) also recorded a high identity of 92.87%. In contrast, considerable homologies were recorded for *T. parva*, *T. lestoquardi*, and *T. orientalis*, from buffalo, sheep, and cattle, respectively. A more evolutionary grouping of the phylogenetic dendrogram was identified for *Theileria* compared with *Babesia* and *Anaplasma*, as all *T. ovis* isolates were on the same tree node, leaving other *Theileria* species, such as *T. parva*, *T. lestoquardi*, and *T. annulata*, on a separate node. A more in-depth look at the dendrogram tree of *Theileria* species revealed that the current *T. ovis* was placed in very close proximity to four *T. ovis* isolates, three from sheep (MN625886.1/Egypt, AY260172.1/Turkey, and MH327772.1/Algeria), and the fourth *A. ovis* from donkeys (MN625903.1/Egypt), respectively. The *T. ovis* of this study was grouped well with other *Theileria* species isolated from other countries and hosts, such as *T. lestoquardi* isolated from goats in India (GenBank accession number MZ220437.1), *T. parva* from water buffalo in Kenya (accession number AF013418.1), and *T. annulata* from cattle in Iraq (accession number MW940543.1). Interestingly, *Theileria* species other than *T. ovis* were from animals other than sheep, including buffalo, cattle, and goats.

## Discussion

Small ruminants are affected by several infectious diseases caused by bacterial, viral, and fungal pathogens, as well as by parasitic diseases caused by protozoan parasites, which have severe negative impacts on their health and welfare. Understanding the epidemiology of these tick-borne blood pathogens is crucial to their effective control and treatment. Thus, the current study involving sheep and goats in the northern coastal region of Egypt, specifically the Matrouh Governorate, had the aim of determining the prevalence of *B. ovis*, *T. ovis*, and *A. ovis* infections and identifying associated risk factors. Molecular detection by PCR using species-specific primers combined with sequencing, BLAST queries, and phylogenetic analysis was applied to detect implicated species.

Utilizing PCR, the total prevalence of the tick-borne blood pathogens *B. ovis*, *T. ovis*, and *A. ovis* was 43%. This was a slightly lower incidence rate than that recorded in Turkey [13], where the PCR indicated the prevalence of these parasites in small ruminants was 68.2%. Similarly, a higher molecular prevalence rate of 69.2% was recorded in sheep and goats in Iraq [14].

The present study detected that there was a significant association between animal sex and the prevalence of tick-borne blood pathogens. This finding concurs with [15], who reported that females were significantly associated with higher infection rates of protozoan blood parasites, and argued that female animals often experienced stress factor-associated immunosuppression, which was primarily associated with pregnancy and lactation, which their male counterparts did not experience. However, in larger ruminants, such as cattle, no significant association between sex and protozoan blood parasite infection has been reported [16].

In this study, the breeding system design was one of the most important risk factors identified. The open breeding system reported a significant association with higher infection rates of tick-borne blood pathogens. Similar evidence was reported in other studies, as [17, 18] demonstrated significant associations between high infection rates and semi-intensive outdoor breeding systems. This is primarily attributed to the higher likelihood of encountering vector-borne disease agents, which is reflected in higher blood parasite infection rates. In a related study, the open breeding system and outdoor grazing were shown to have increased chances of exposure to several vectors, including hard ticks, which are the primary vectors of protozoan blood parasite transmission [19]. Moreover, the open breeding system is associated with climatic changes (fluctuating weather conditions) that, through their effects, affect animal immunity.

The prevalence of *A. ovis* in sheep and goats revealed by PCR was 24%. Similarly, [20] in Iran, and [21] in Medina, Saudi Arabia, reported that the prevalence of *A. ovis* among small ruminants according to PCR was 27.5% and 25.3%, respectively. In contrast, a higher *A. ovis* prevalence rate of 60.89% according to PCR was reported in 12 Chinese provinces, which is nearly double the prevalence rate seen in the present study [22].

PCR amplification of the 16 S rRNA gene of *Anaplasma* species yielded an amplicon of ~ 870 bp. This is as expected for the amplified product specific to *Anaplasma* species, as reported by another study in the West Azerbaijan province, Iran, in which the targeting of the same gene produced a closely related size amplicon and sequence of 866 bp [23]. BLAST-homology queries unsurprisingly produced hits that were all identified as *A. ovis* homologues from different animal species, mostly sheep, but also from other animal hosts such as cattle, deer, and ticks. BLAST analysis results were further confirmed by phylogenetic analysis and its dendrogram tree as *A. ovis* in the current study. It was placed in the same clade with *A. ovis* isolated from goats from Iran, and *A. ovis* isolated from sheep from Serbia and Russia under accession numbers EU92511.1, GQ92510.1, and MT062868.1, respectively. These were in close evolutionary proximity to *A. ovis* from sheep, ticks, humans, and deer from Turkey, China, Cyprus, and Spain, respectively. Although not in the same clade (node of branching), the current *A. ovis* isolate relates to other species of *Anaplasma*, such as *A. bovis* isolated from deer in Japan (GenBank accession number AB211163.1), cattle in China (GenBank accession number MH255937.1), and ticks in China (GenBank accession number MN044717.1). It also relates to *A. centrale* isolated from cattle from Kyrgyzstan and South Africa with accession numbers MW672119.1, and GQ130291.1, respectively, as well as *A. marginale* isolated from cattle from China (GenBank accession number AJ63304.1), ticks from Pakistan (GenBank accession number ON52757.1), and *A. phagocytophilum* identified in rats and ticks from Canada and China, respectively.

The prevalence rate for *Babesia* infections according to PCR in this study is consistent with PCR-based molecular prevalence rates of *Babesia* infection recorded in two studies in Iran and Greece, which reported 16.7% and 15%, respectively [24, 25]. However, [26] in an independent study in the North Khorasan province of Iran, researchers reported a lower prevalence rate of 6.6% for *B. ovis* infection.

The amplification of the 18 S rRNA gene using *Babesia*-specific primers yielded a band at ~ 550 bp, which corresponds well with the same results of DNA amplification by the same primers as recorded by [27] in Elazig province, Turkey. Phylogenetic and BLAST analysis of the 18 S rRNA sequence of our *Babesia* isolate revealed that it was *B. ovis*, which was recorded in GenBank with accession number MZ801782.1.

These *B. ovis* isolates showed high identity (> 94%) with *B. ovis* isolates from other countries, including Uganda, Turkey, and Spain (GenBank accession numbers MT114713.1, MG569902.1, and AY150058.1, respectively), and were isolated from goats, horses, and goats, respectively. High homologous sequences from *Babesia* spp. other than *B. ovis* were retrieved and were highly identified (> 90%) with the current isolate. These included *B. orientalis*, *B. bigemina*, and *B. divergens*, from ticks (China), buffalo (Iraq), and cattle (Ireland), respectively.

In addition to *B. orientalis*, all *B. ovis* sequences were clustered together on the same node of the evolutionary dendrogram, including isolates from sheep, goats, horses, and ticks. For *B. orientalis*, this is not surprising, as until recently it had not been registered as a separate species due to the unusually high number of shared identities with other commonly known *Babesia* spp. [28].

More divergent *Babesia* species, such as *B. bigemina*, *B. divergens*, and *B. bovis*, are clustered on branches separate from *B. ovis*. When compared with *Anaplasma*, a more evolutionary conserved relationship was detected for various *B. ovis* isolates. The prevalence of *Theileria* according to PCR was 17% in the current study. Similar findings in Uganda were reported by [29], with an overall infection rate of 13.4%. In contrast, [30] recorded a higher prevalence of *Theileria* in different districts in China. Additionally, a high incidence rate of 66.25% was recorded for theileriosis in small ruminants in Kenya [31]. Lastly, PCR targeting the 18 S rRNA of *Theileria* species yielded an amplicon of 500 bp, an expected PCR product size as amplified by the same *Theileria*-specific primers on blood samples collected from small ruminants in China [32].

## Conclusion

In our current study, the data revealed that the PCR, combined with sequencing and phylogenetic analysis was successfully applied in detecting two of the most important tick-borne protozoan and rickettsial pathogens that are circulating in populations of small ruminants on the North Coast of Egypt. Molecular phylogenetic analysis revealed significant identities with corresponding homologous isolates from Asia, Africa, and Europe. Analysis of risk factors showed that the grazing system is a crucial determinant of the propagation of vector-borne pathogens and that every effort should be directed toward changing the open grazing habits of small ruminant herds into a more closed system. The next study concerning the current topic should essentially be directed toward surveying small ruminants in a more extended geographical manner to develop an epidemiological map of tick-borne protozoon and rickettsial infective organisms in small ruminants in Egypt. Finally, more efforts are required to develop more effective measures to control blood parasites and, subsequently, optimize small animal health and productivity in Egypt.

## Materials and methods

### Study settings

This study was conducted in the northern coastal region of Egypt, with the main focus on Matrouh Governorate (31.352778°N, 27.236111°E), which included four districts: Elhammam, Marsa-Matrouh, Eldabaa, and El-Alamein. Herds of small ruminants are distributed throughout the four geographical regions. The study utilized a cross-sectional design. In this cross-sectional study, sheep and goats were sampled without regard to disease status and studied at a particular point in time. All methods were carried out following relevant guidelines and regulations. Ethical approval for this study was obtained from the scientific research ethical committee of the Faculty of Veterinary Medicine, Alexandria University (Approval #00012098).

### Sample size

One hundred blood samples were collected from 76 sheep and 24 goats. The sample size was determined based on the total capital herd population of small ruminants in the designated four out of eight districts in Matrouh Governorate. The total was 178,408 heads, according to the latest data from the Matrouh Governorate Directorate of Veterinary Medicine (**Access Link 1)**. According to a previous study of blood protozoan parasitic infection, which indicated a prevalence rate of 10% [33], the minimum sample size for this study was 98 heads, with a margin of error of 5% and a confidence level of 90%. Where the sample size was determined by using the Sample Size Calculator by Raosoft, Inc., http://www.raosoft.com/samplesize.html with a confidence level of 90%. The samples were collected from four districts. Within each district, by using the EPI method (bottle spinning) https://academic.oup.com/ije/article to determine a random direction to select random farms along the chosen direction pointing outwards from the centre of the district to its boundary, then within all farms selected, the total sheep and goats were required to be enrolled in the current study by a random systematic method. https://study.com/academy/lesson/systematic-random-samples.

### Sampling technique

A multistage sampling technique was applied as follows: an unequal probability of selection with unequal-sized clusters was utilized during the first stage, and proportional allocation according to cluster size was used during the second stage to select 76 sheep and 24 goats from the four districts. From each of the selected animals, 2 ml of blood was collected from the jugular vein into anticoagulant EDTA (Ethylenediaminetetraacetic acid)-coated vacutainer tubes (Thermo Scientific™, Thermo Fisher Scientific Inc., Waltham, MA, USA). These samples were stored at − 20 ºC until further processed for microscopy and DNA extraction. In parallel to the sample collection, the body condition scoring was performed on the sampled animals following the technical body condition scoring protocol that was developed at Langston University (**Access Link 2**), which considered the status of the lumbar region, the rib cage, and the sternum, while examining the animal. Animals that scored ≥ 3 were considered high scores, while those ≤ 3 were recorded as having low body conditions. The animal temperature was also recorded, and the body temperature was considered normal or febrile (feverish) concerning the normal range of the body temperature of sheep and goats (sheep: 38.3–39.9 °C; goat: 38.5–39.7 °C; in the Merck Veterinary Manual 20th Edition).

### Microscopic examination

For each sample, a thin blood smear was prepared on a glass slide, air-dried, and fixed for 5 min in methyl alcohol. The fixed blood smears were then stained with 5% Giemsa stain for 30 min. The Giemsa-stained blood smears were washed gently with tap water to remove the excess stain, and the stained films were examined using oil immersion lenses (1000x magnification) of a light microscope [34]. To set up a blindfolded examination strategy, three smears were prepared from each blood sample, and these were examined by two independent researchers, setting up the examination of up to six technical replicates from each sample, to conclude whether the sample was positive or negative.

### DNA extraction and PCR-based amplification

Total genomic DNA was extracted from whole blood samples using the Gene JET Whole Blood Genomic DNA Purification Mini Kit (Thermo Fisher Scientific Baltics, UAB, Lithuania), as directed by the manufacturer. For the PCR, three sets of primer pairs were designed to amplify species-specific targets of *Babesia ovis*, *Theileria ovis*, and *Anaplasma ovis* (Table 6). For *B. ovis* and *T. ovis*, the primers were designed to specifically amplify targets from the 18 S rRNA sequences as previously specified [35], while for *A. ovis*, the primers were designed to specifically amplify targets from the major surface protein 4 (msp4) gene [36].

**Table 6:** Oligo primer sequences used in the current study

Parasite	Primer sequence (5′-3′)	Target	Amp. size	Ref.
*Babesia* spp.	F: 5-TGGGCAGGACC TTGGTTCTTCT-3 R: 5-CCGCGTAGCGCCGGCTAAATA-3	18 S rRNA	549 bp	[33]
*Theileria* spp.	F: 5-TCGAGACCTTCGGGTGGCGT-3 R: 5-TCCGGACATTGTAAAACAAA-3	18 S rRNA	509 bp	[33]
*Anaplasma* spp.	F: 5-GGGAGCTCCTATGAATTACAGAGAATTGTTTAC-3 R: 5-CCGGATCCTTAGCTGAACAGGAATCTTGC-3	Msp4	870 bp	[34]

### DNA sequencing and analysis

To establish the molecular identities of the amplified PCR products, representative three samples that were positive (by microscopy and PCR) for the three pathogens (single sample per pathogen), and that also corresponded to the expected (annotated) molecular masses for each primer set, were selected for DNA sequencing. Selected PCR amplicons were sequenced in a single-sense stranded direction with the sample PCR sense primer, and by using the ABI 3500 Applied Biosystems genetic analyzer (Applied Biosystems, CA, USA). Assembled sequences (between 509 and 1345 bp) were manually screened and curated to remove non-allocated poly-N nucleotides (N). Curated clean sequences were queried in the standard nucleotide BLAST (BLASTn) search tool of the National Center for Biotechnology Information (NCBI, USA) (https://blast.ncbi.nlm.nih.gov/Blast.cgi) [37].

Throughout all the BLASTn queries, default settings were applied, except for the use of the “more divergent sequences” option instead of “closely related sequences” in the BLASTn search platform. The top sequence hits resulting from the BLASTn analysis showed significant similarities and identities with the sequences from the current study that were selected. Reverse-BLASTn using individual sequences was performed to confirm the molecular identities of the sequenced samples versus previously published sequence data deposited at GenBank (https://www.ncbi.nlm.nih.gov/genbank/) as reference sequences. Data corresponding to selected sequence hits, including names, accession numbers, animal hosts, countries, homology sequence identities (%), percentage query coverages (%), and E. values were extracted from BLAST and GenBank data and were correspondingly tabulated for each of the three blood pathogens. Finally, sequence data from highly homologous isolates was downloaded and used in the phylogenetic analyses.

### Phylogenetic analysis

All extracted sequences, including the current ones, were subjected to sequence alignment, with two approaches applied. First, the BLAST sequence alignment for each of the three BLASTn outputs was downloaded, scanned, and curated manually to remove non-allocated gaps and to delete extensions at both ends of the aligned sequence data. In the second approach, homologous sequences were extracted from the GenBank followed by the use of the MEGA v.7 platforms for sequence alignment. The aligned sequences were then entered into the phylogenetic analysis within MEGA v.7 software [38]. Evolutionary relationships between different species of each of the three tick-borne blood pathogens were inferred from the analysis results by using the Saitou-Nei Neighbor-Joining (NJ) method with bootstrapping set at 1000 replications in MEGA v.7 [39].

### Statistical analysis

Data were statistically analyzed using IBM SPSS Statistics for Windows, version 22 (IBM Corp., Armonk, NY, USA), and by applying Pearson’s Chi-squared test to determine the statistical significance and associations between prevalence rates and risk factors as independent variables. The confidence interval was set at level 95% and the odds ratio (OR) to predict the associations between infection rates and risk factors were considered significant at *P* < 0.05.

## Data Availability

The datasets generated and/or analyzed during the current study are available in the GenBank of the National Center for Biotechnology Information (GenBank-NCBI) repository, which can be readily accessed by applying the following accession numbers: MZ801782.1, MZ801783.1, and MZ889099.

## Declarations

### Guidelines

All methods were carried out in accordance with relevant guidelines and regulations.

### ARRIVE guidelines

The authors confirm that the study was carried out in compliance with the ARRIVE guidelines.

## References

[1] Ali S, Zhao Z, Zhen G, Kang JZ, Yi PZ (2019). Reproductive problems in small ruminants (sheep and goats): a substantial economic loss in the world. Large Anim Rev.

[2] Bello AM, Lawal JR, Dauda J, Wakil Y, Mshellia ES, Abubakar MI, Biu AA (2017). Prevalence of haemoparasites in Balami sheep from Maiduguri, northeastern Nigeria. Direct Res J Vet Med Anim Sci.

[3] Li Y, Galon EM, Guo Q, Rizk MA, Moumouni PFA, Liu M, Li J, Ji S, Chahan B, Xuan X (2020). Molecular detection and identification of Babesia spp., Theileria spp., and Anaplasma spp. in sheep from border regions, northwestern China. Front Vet Sci.

[4] Kasozi KI, Namayanja M, Gaithuma AK, Mahero M, Matovu E, Yamagishi J, Sugimoto C, MacLeod E (2019). Prevalence of hemoprotozoan parasites in small ruminants along a human-livestock-wildlife interface in western Uganda. Vet Parasitol Reg Stud Reports.

[5] Wang J, Gao S, Zhang S, He X, Liu J, Liu A, Li Y, Liu G, Luo J, Guan G, Yin H (2020). Rapid detection of *Babesia motasi* responsible for human babesiosis by cross-priming amplification combined with a vertical flow. Parasit Vectors.

[6] Alanazi AD, Said AE, Ghoneim AM, Alyousif MS, Alanazi IO (2019). A comprehensive evaluation and first molecular report of *Theileria ovis* infection in small ruminants in Saudi Arabia. Trop Anim Health Prod.

[7] Cabezas-Cruz A, Gallois M, Fontugne M, Allain E, Denoual M, Moutailler S, Devillers E, Zientara S, Memmi M, Chauvin A, Agoulon A, Vayssier-Taussat M, Chartier C (2019). Epidemiology and genetic diversity of *Anaplasma ovis* in goats in Corsica, France. Parasites Vectors.

[8] Kasozi KI, Welburn SC, Batiha GES, Marraiki N, Nalumenya DP, Namayanja M, Matama K, Zalwango KK, Matovu W, Zirintunda G, Ekou J, Kembabazi S, Mugasa CM, Kitibwa A, Tayebwa DS, Musinguzi SP, Mahero M, Ssengendo I, Nanteza A, Matovu E, MacLeod ET (2021). Molecular epidemiology of anaplasmosis in small ruminants along a human-livestock-wildlife interface in Uganda. Heliyon.

[9] Belkahia H, Ben Said M, El Mabrouk N, Saidani M, Cherni C, Ben Hassen M, Bouattour A, Messadi L (2017). Seasonal dynamics, spatial distribution and genetic analysis of *Anaplasma* species infecting small ruminants from Northern Tunisia. Infect Genet Evol.

[10] Orkun Ö, Çakmak A, Nalbantoğlu S, Karaer Z (2019). Molecular detection of a novel *Babesia* sp. and pathogenic spotted fever group *rickettsiae* in ticks collected from hedgehogs in Turkey: Haemaphysalis erinacei, a novel candidate vector for the genus *Babesia*. Infect Genet Evol.

[11] Ceylan O, Xuan X, Sevinc F (2021). Primary tick-borne protozoan and rickettsial infections of animals in Turkey. Pathog.

[12] Menshawy SM (2020). A review on bovine babesiosis in Egypt. EVMSPJ.

[13] Zhou M, Cao S, Sevinc F, Sevinc M, Ceylan O, Ekici S, Jirapattharasate C, Moumouni PFA, Liu M, Wang G, Iguchi A, Vudriko P, Suzuki H, Xuan X (2017). Molecular detection and genetic characterization of *Babesia*, *Theileria* and *Anaplasma* amongst apparently healthy sheep and goats in the central region of Turkey. Ticks Tick Borne Dis.

[14] Renneker S, Abdo J, Bakheit MA, Kullmann B, Beyer D, Ahmed J, Seitzer U (2013). Coinfection of sheep with *Anaplasma*, *Theileria* and *Babesia* species in the Kurdistan region. Iraq Transbound Emerg Dis.

[15] Benedicto B, Ceylan O, Moumouni PFA, Lee SH, Tumwebaze MA, Li J, Galon EM, Liu M, Li Y, Ji S, Ringo A, Rizk M, Sevinc F, Xuan X (2020). Molecular detection and assessment of risk factors for tick-borne diseases in sheep and goats from Turkey. Acta Parasitol.

[16] Abdela N, Ibrahim N, Begna F (2018). Prevalence, risk factors and vectors identification of bovine anaplasmosis and babesiosis in and around Jimma town, Southwestern Ethiopia. Acta Trop.

[17] Egbe-Nwiyi TN, Sherrif GA, Paul BT (2018). Prevalence of tick-borne haemoparasitic diseases (TBHDS) and haematological changes in sheep and goats in Maiduguri abattoir. J Vet Med Anim Health.

[18] Kabir MHB, Mondal MMH, Eliyas M, Mannan MA, Hashem MA, Debnath NC, Miazi OF, Kashem MA, Islam MR, Elahi MF (2011). An epidemiological survey on investigation of tick infestation in cattle at Chittagong District, Bangladesh. Afr J Microbiol Res.

[19] Muhanguzi D, Matovu E, Waiswa C (2010). Prevalence and characterization of *Theileria* and *Babesia species* in cattle under different husbandry systems in western Uganda. Int J Anim Vet Adv.

[20] Yousefi A, Rahbari S, Shayan P, Sadeghi-Dehkordi Z, Bahonar A (2017). Molecular detection of *Anaplasma marginale* and *Anaplasma ovis* in sheep and goat in west highland pasture of Iran. Asian Pac J Trop Biomed.

[21] Shabana II, Alhadlag NM, Zaraket H (2018). Diagnostic tools of caprine and ovine anaplasmosis: a direct comparative study. BMC Vet Res.

[22] Qiu H, Kelly PJ, Zhang J, Luo Q, Yang Y, Mao Y, Yang Z, Li J, Wu H, Wang C. Molecular detection of *Anaplasma spp* and *Ehrlichia spp*. in ruminants from twelve provinces of China. Can J Infect Dis Med Microbiol. 2016;9183861. 10.1155/2016/9183861. 10.1155/2016/9183861 PMC5206432 28096822

[23] Noaman V, Bastani D (2016). Molecular study on infection rates of *Anaplasma ovis* and *Anaplasma marginale* in sheep and cattle in West-Azerbaijan province. Iran Vet Res Forum.

[24] Esmaeilnejad B, Tavassoli M, Asri-Rezaei S, Dalir-Naghadeh B, Mardani K, Jalilzadeh-Amin G, Golabi M, Arjmand J. PCR-based detection of *Babesia ovis* in *Rhipicephalus bursa* and small ruminants. J Parasitol Res. 2014;294704. 10.1155/2014/294704. 10.1155/2014/294704 PMC4020301 24876944

[25] Theodoropoulos G, Gazouli M, Ikonomopoulos JA, Kantzoura V, Kominakis A (2006). Determination of prevalence and risk factors of infection with *Babesia* in small ruminants from Greece by polymerase chain reaction amplification. Vet Parasitol.

[26] Seidabadi M, Razmi G, Naghibi A (2014). Molecular detection of *Babesia* spp. in sheep and vector ticks in North Khorasan Province, Iran. Iran J Vet Med.

[27] Altay K, Dumanli N, Holman PJ, Aktas M (2005). Detection of *Theileria ovis* in naturally infected sheep by nested PCR. Vet Parasitol.

[28] He L, Liu Q, Yao B, Zhou Y, Hu M, Fang R, Zhao JA (2017). Historical overview of research on *Babesia orientalis*, a protozoan parasite infecting water buffalo. Front Microbiol.

[29] Tumwebaze MA, Byamukama B, Tayebwa DS, Byaruhanga J, Angwe MK, Galon EM, Liu M, Lee SH, Ringo AE, Adjou Moumouni PF, Li J, Li Y, Ji S, Vudriko P, Xuan X (2020). First molecular detection of Babesia ovis, Theileria spp., Anaplasma spp., and Ehrlichia ruminantium in goats from western Uganda. Pathogens.

[30] Cao S, Zhang S, Jia L, Xue S, Yu L, Kamyingkird K, Moumouni PFA, Moussa AAEM, Zhou M, Zhang Y, Terkawi MA, Masatani T, Nishikawa Y, Xuan X (2013). Molecular detection of *Theileria* species in sheep from Northern China. J Vet Med Sci.

[31] Ghai RR, Mutinda M, Ezenwa VO (2016). Limited sharing of tick-borne hemoparasites between sympatric wild and domestic ungulates. Vet Parasitol.

[32] Li Y, Guan G, Ma M, Liu J, Ren Q, Luo J, Yin H (2011). *Theileria ovis* discovered in China. Exp Parasitol.

[33] Durrani S, Khan Z, Khattak RM, Andleeb M, Ali M, Hameed H, Taqddas A, Faryal M, Kiran S, Anwar H, Riaz M (2012). A comparison of the presence of *Theileria ovis* by PCR amplification of their SSU rRNA gene in small ruminants from two provinces of Pakistan. Asian Pac J Trop Med.

[34] Kohli S, Atheya UK, Thapliyal A (2014). Prevalence of theileriosis in cross-bred cattle: its detection through blood smear examination and polymerase chain reaction in Dehradun district, Uttarakhand, India. Vet World.

[35] Aktaş M, Altay K, Dumanlı N (2005). Development of a polymerase chain reaction method for diagnosis of *Babesia ovis* infection in sheep and goats. Vet Parasitol.

[36] De La Fuente J, Torina A, Caracappa S, Tumino G, Furlá R, Almazán C, Kocan KM (2005). Serologic and molecular characterization of Anaplasma species infection in farm animals and ticks from Sicily. Vet Parasitol.

[37] Altschul SF, Gish W, Miller W, Myers EW, Lipman DJ (1990). Basic local alignment search tool. J Mol Biol.

[38] Kumar S, Stecher G, Tamura K (2016). MEGA7: molecular evolutionary genetics analysis version 7.0 for bigger datasets. Mol Biol Evol.

[39] Saitou N, Nei M, July I. The neighbor-joining method: a new method for reconstructing phylogenetic trees. Mol Biol Evol. 1987;4(4): 406–425. 10.1093/oxfordjournals.molbev.a040454. PMID: 3447015. 10.1093/oxfordjournals.molbev.a040454 3447015

